# Escape the EM Boards: Interactive Virtual Escape Room for GI Board Review

**DOI:** 10.21980/J8H63F

**Published:** 2021-04-19

**Authors:** Megan Gillespie

**Affiliations:** * Jefferson Health - Northeast, Department of Emergency Medicine, Philadelphia, PA

## Abstract

**Audience:**

This interactive and entirely virtual escape room based on high yield gastrointestinal/abdominal board review material is a didactic activity for emergency medicine residents of all postgraduate years and third- or fourth-year medical students. This escape room can be completed in small teams or as individuals in a remote, in-person, or mixed location setting.

**Introduction:**

2020 is a year that will forever change medical education. The novel coronavirus 2019 pandemic caused many national, state, local, or hospital specific recommendations for transition of meetings to be completely virtual or to only allow for limited in-person meetings. In order to abide by these recommendations, the majority of medical education transitioned to online platforms. Now more than ever, creative and engaging methods for expanding clinical knowledge and teaching teamwork as well as unique integrations of technology for medical education delivery are needed.

**Educational Objectives:**

By the end of this didactic activity, learners will be able to:

1. Identify causes of upper gastrointestinal bleeding.
2. Recall test-taking buzzwords for infectious causes of diarrhea.
3. Acknowledge the correct hepatitis B titers that correspond with various clinical scenarios.
4. Describe the management for alkali caustic ingestions.
5. Determine the components of Maddrey Discriminant Function Score, Charcot’s triad, Ranson’s Criteria for Pancreatitis, and Glasgow-Blatchford Score.
6. Diagnose specific gastrointestinal diseases from a clinical description.
7. Choose the correct gastrointestinal diagnosis based on clinical image findings.
8. Demonstrate teamwork in solving problems.

**Educational Methods:**

“Gamification” is the use of game elements in a nongame context.^1^ Gamification creates active, engaged learning opportunities and so it is a highly favored educational method for millennial learners.^1^ An Escape Room is a team-based game where a small group is locked in a room and they pick up objects at random in this room to solve a series of clues that will play a role in solving the final clue to “escape” the locked room. This didactic learning activity utilizes technology to deliver a fun and interactive distance learning activity that resembles a live escape room.

The interactive virtual escape room provided is a no cost, unique alternative educational activity that can be done either entirely or partly remotely. In order to replicate this team-based didactic exercise, the instructor needs to simply divide residents and students into small groups and then share with each group the link provided.

**Research Methods:**

A five-point Likert scale survey was distributed to participating learners after completing this activity to assess relevance and satisfaction of the high yield gastrointestinal/abdominal board review escape room. Thirty-two of forty-two participating residents/medical students completed this seven-question survey.

**Results:**

All participating resident/medical students who completed the survey enjoyed this activity and that this was a unique learning experience, and the majority of participating residents/medical students thought this interactive virtual escape room was easy to use, learned something, thought this was a helpful way to review for emergency medicine boards or in-training exams, and preferred a game like this to a standard lecture. Additionally, over half of survey respondents said they are going to try to come up with a game for their assigned board review presentations.

**Discussion:**

Escape room gamification of high yield gastrointestinal/abdominal board review material was an engaging, fun, and effective distance learning activity for residents and medical students. This didactic activity not only promoted interactive learning but also encouraged virtual teamwork among small groups.

**Topics:**

Gastroenterology, GI, abdominal, board review, in-training exam review, escape room, virtual didactics, distance learning, high yield, upper GI bleeding, esophageal varices, diarrhea, alkali ingestion, volvulus, foreign body, intussusception.

## USER GUIDE

[Table t1-jetem-6-2-sg8]

**Table t1-jetem-6-2-sg8:** 

**List of Resources:**	
Abstract	8
User Guide	10
Small Group Materials	14
Appendix A: Clue Answers and Master Sheet	14
Appendix B: GI Review Study Outline for Interactive Virtual Escape Room	19

**Learner Audience:**

Medical Students, Interns, Junior Residents, Senior Residents

**Time Required for Implementation:**

50 Minutes total: 10 minutes to explain this virtual interactive escape room and divide into small teams, 40 minutes for learners to complete the escape room.

**Recommended Number of Learners per Instructor**:

It is recommended that learners are pre-sorted into groups of 4–6 residents of various post-graduate years and 1–2 medical students per team so that teams are small enough that everyone can participate. One instructor could provide requested support to all groups simultaneously, or there can be one facilitator assigned to each small group in case the team gets stuck on a particular clue.

**Topics:**

Gastroenterology, GI; abdominal, board review, in-training exam review, escape room, virtual didactics, distance learning, high yield, upper GI bleeding, esophageal varices, diarrhea, alkali ingestion, volvulus, foreign body, intussusception.

**Objectives:**

By the end of this didactic activity, learners will be able to:

1. Identify causes of upper gastrointestinal bleeding.
2. Recall test-taking buzzwords for infectious causes of diarrhea.
3. Acknowledge the correct hepatitis B titers that correspond with various clinical scenarios.
4. Describe the management for alkali caustic ingestions.
5. Determine the components of Maddrey Discriminant Function Score, Charcot’s triad, Ranson’s Criteria for Pancreatitis, and Glasgow-Blatchford Score.
6. Diagnose specific gastrointestinal diseases from a clinical description.
7. Choose the correct gastrointestinal diagnosis based on clinical image findings.

### Linked objectives and methods

Gamification and technology-enhanced active learning for medical education are the two main conceptual frameworks utilized in creating this interactive and virtual escape room based on high yield gastrointestinal/abdominal board review material.^6^,^7^

### Recommended pre-reading for facilitator

**Figure 1:**
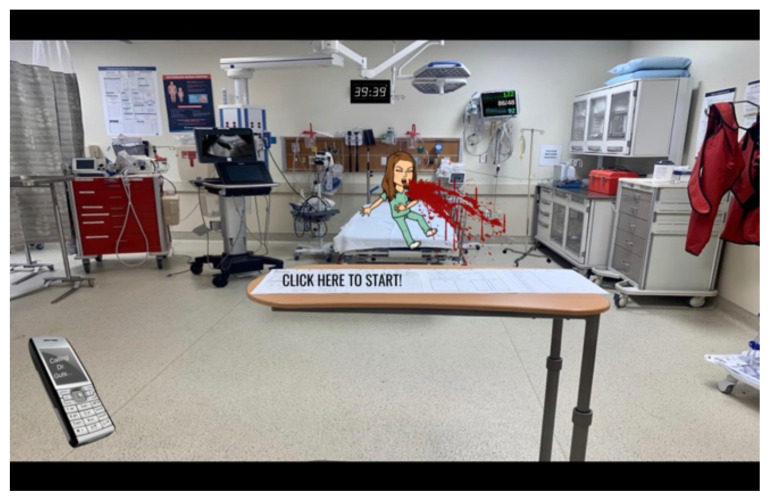
Interactive, virtual escape room for GI board review, created on Google slides. Permanent web address for virtual escape room is bit.ly/giescaperoom.

**Figure 2:**
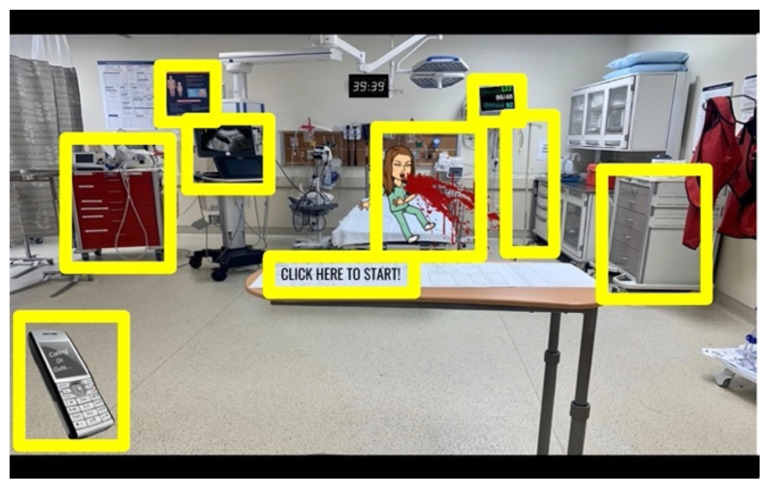
Interactive, virtual escape room for GI board review, created on Google slides, with clickable link locations identified for escape room and patient information, six clues, and one final clue.

The Clue Master Document, supplemental file, details how the escape room works and where clues are located in the escape room and the answers to these clues. It is also recommended that the facilitator trial the escape room link themselves (available at bit.ly/giescaperoom) to familiarize themselves with where the patient information, six clues, and final clues are linked within the virtual escape room (Figure 1, 2). Please note that although this escape room could be used on a tablet or phone, it is optimal to use this virtual escape room on a laptop or desktop because each clue that is clicked will open a new tab and you will need to click back to the escape room tab after completing clues. Additionally, if further information is desired, the GI Study Guide, supplemental file, is a thorough high yield gastrointestinal study outline compiled from Dr. Carol Rivers' Written Board Review resources via Ohio ACEP app, “The Ultimate Emergency Medicine Guide: The only EM book you need to succeed,” by Dr. Sajid Khan, Rosh Review Question Bank, and Hippo EM Board Review Videos.^2^–^5^

### Associated Materials

- Appendix A: Clue Answers and Master Sheet
- Appendix B: GI Review Study Outline for Interactive Virtual Escape Room

### Link to Escape the EM Boards: Interactive Virtual Escape Room for GI Board Review

- https://bit.ly/giescaperoom

### Results and tips for successful implementation

**Figure 3:**
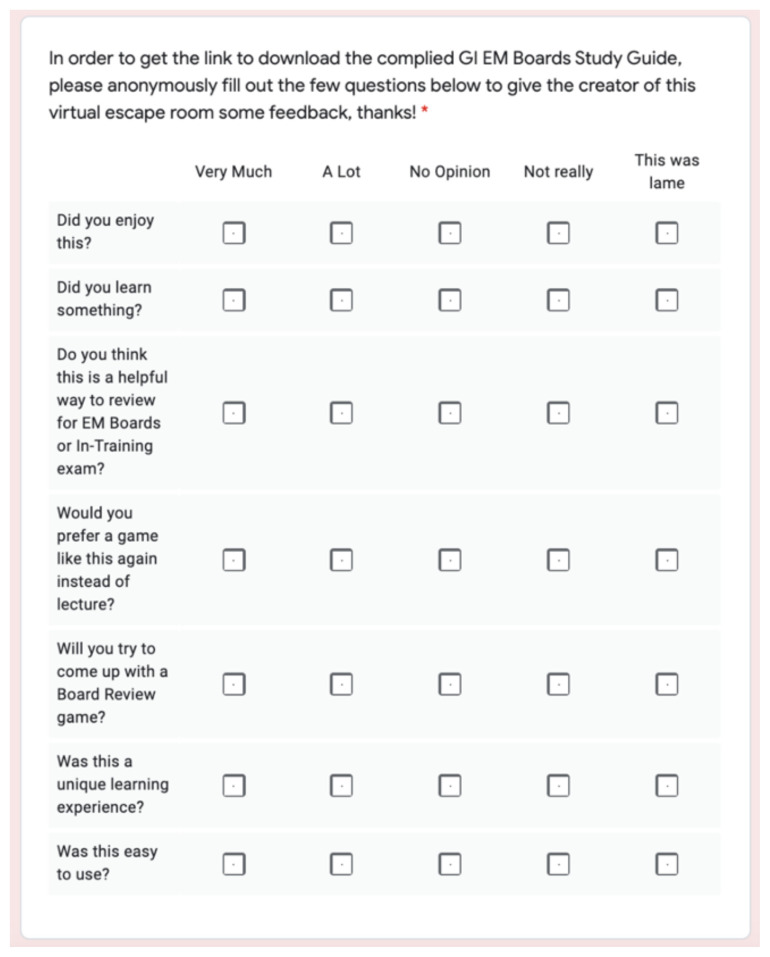
Post escape room survey questions and Likert scale.

This interactive virtual escape room was trialed on individual residents and faculty members prior to using this didactic exercise on forty-two total residents and medical students during a weekly didactic conference. There were thirty-five residents and seven medical students that participated in this virtual escape room, and thirty-two of these forty-two total residents/medical students completed the post escape room survey. This post escape room survey consisted of a five-point Likert scale to answer seven questions to assess relevance and satisfaction of the high yield gastrointestinal/abdominal board review escape room. The Likert scale options included “very much,” “a lot,” “no opinion,” “not really,” and “this was lame” as options and in this order represented strongly agree to strongly disagree (Figure 3).

Of the thirty-two survey respondents, 100% said they “very much” enjoyed this activity and that this was a unique learning experience. Twenty-eight of thirty-two, or 87.5%, said they “very much” thought this interactive virtual escape room was easy to use. Twenty-four of thirty-two, or 75%, “very much” learned something, thought this was a helpful way to review for emergency medicine boards or in-training exams, and preferred a game like this to a standard lecture. Nineteen of thirty-two, 59.4% of survey respondents said they are “very much” going to try to come up with a game for their assigned board review presentations.

**Figure 4:**
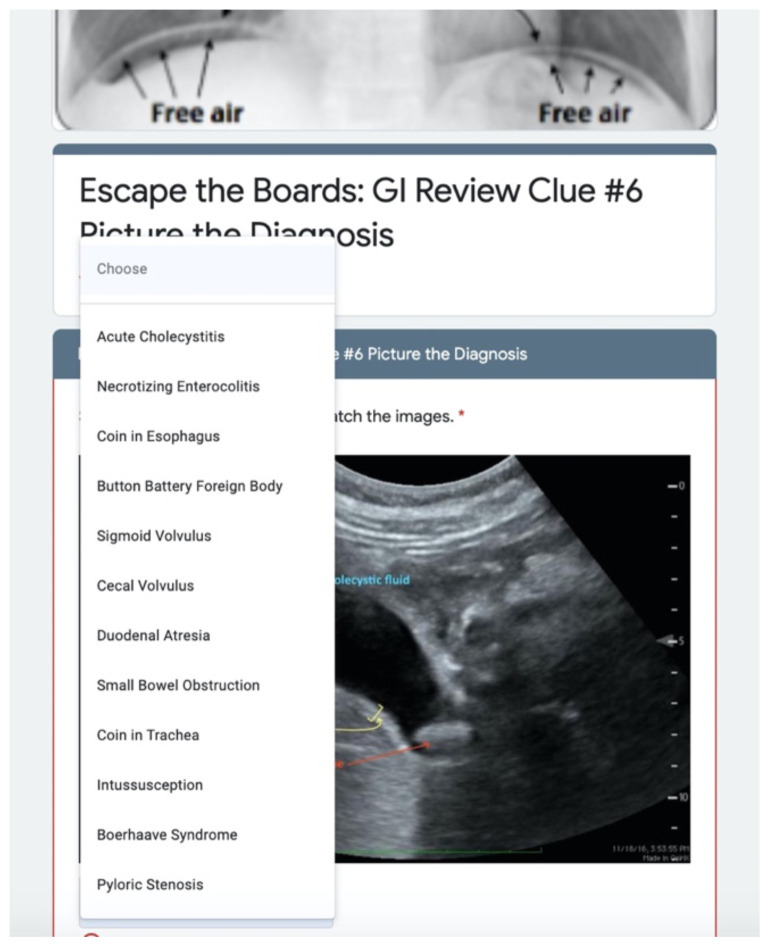
Embedded linked clues open Google Forms to collect answers to clues.

**Figure 5:**
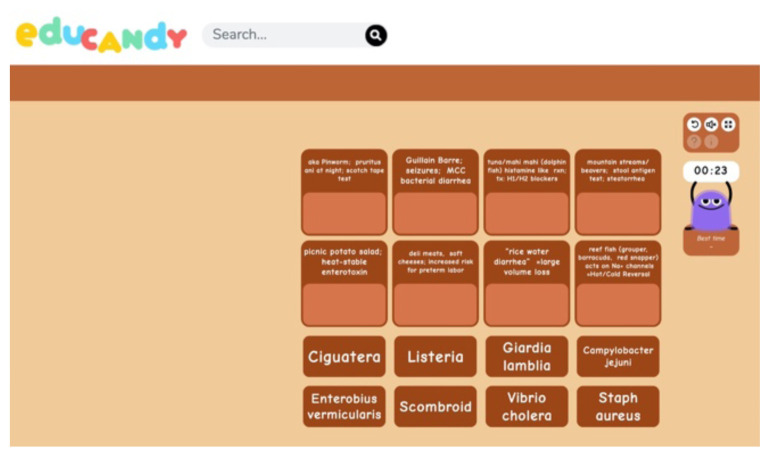
One of the linked clues takes participants to Educandy.com to complete a matching game based on high yield test diarrhea buzzwords.

**Figure 6:**
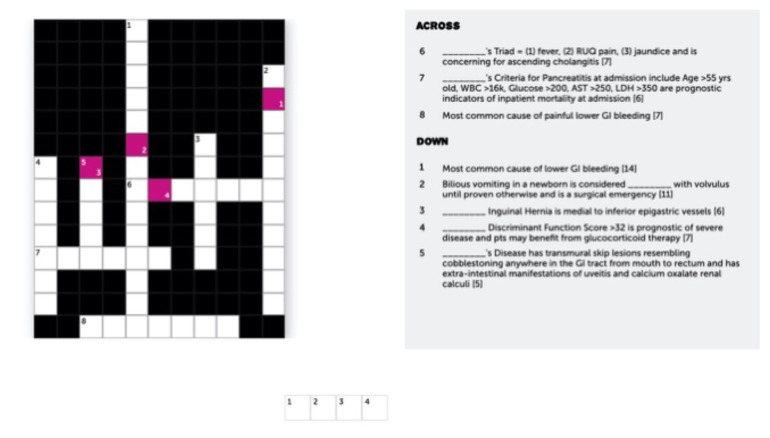
One of the linked clues takes participants to Puzzel.org to complete a crossword based on high yield test gastrointestinal clinical descriptions.

**Figure 7:**
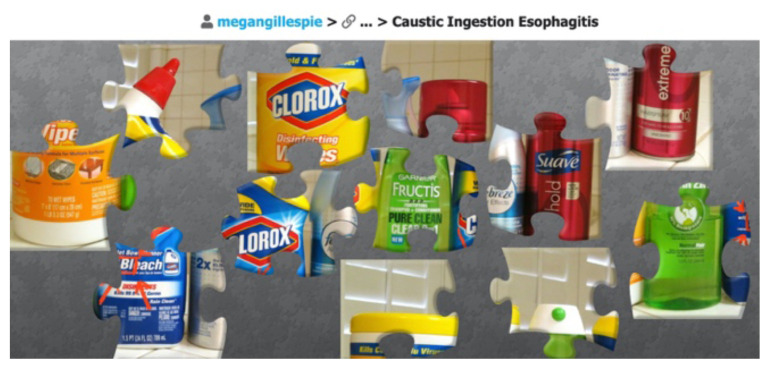
One of the linked clues takes participants to Jigsawplanet.com to complete a puzzle to determine a caustic agent that was ingested.

In terms of tips for successful utilization of this interactive virtual escape room, participating residents/medical students should have access to a laptop or desktop. Please note that although this escape room could be used on a tablet or phone, it is optimal and easier to use this virtual escape room on a laptop or desktop because each clue that is clicked will open a new tab and you will need to click back to the escape room tab after completing clues. It took all of the individual or group participants 30–35 minutes to complete so it is recommended to allow for 40 minutes to ensure completion by all participants. It is also recommended to allow for 10 minutes before starting the escape room to explain this didactic activity. It should be explained that participants will be divided into small teams via Zoom break out rooms (or similar alternative video conference tool), a link will be provided which will be a photo of a resuscitation bay that has links embedded in certain aspects of the photo that the teams will have to click on to find patient information, six clues, and a final clue that uses information provided from solving the previous six clues to successfully “escape the room.” The easiest way for the small group break outs to work together is to designate one person of the group to share their screen and be the clicker/typer as the team works their way through the escape room clues (Figures 4–7). Of note, each clue clicked will open a new tab; to get back to the escape room, participants simply need to click back to the web browser tab with the escape room. If teams are stuck, the facilitator, or “clue master,” who is already familiar with the escape room or has the provided clue master document can give clues if needed (this was not necessary with the trials noted here). The link, bit.ly/giescaperoom, should be provided to all participants by email or video conference chat just prior to starting this didactic exercise so teams can start at a similar time to see who can successfully escape the quickest.

The facilitator should in advance divide participants into groups of 4–6 residents of various post-graduate years and 1–2 medical students per team so that teams are small enough that everyone can participate, and the teams are roughly even in terms of medical knowledge. This didactic activity can be performed completely virtually or in-person or as a mix of in-person and virtual settings. Participants will need access to a laptop/computer regardless of setting to be able to utilize the escape room and video conference to work as part of a team.

Upon completion of the escape room, participants who escape successfully by solving all clues, including the final clue, are rewarded with a high yield gastrointestinal study outline compiled from Dr. Carol Rivers' Written Board Review resources via Ohio ACEP app, “The Ultimate Emergency Medicine Guide: The only EM book you need to succeed,” by Dr. Sajid Khan, Rosh Review Question Bank, and Hippo EM Board Review Videos. This study guide should be emailed along with the link for the escape room (bit.ly/giescaperoom, the link does not expire) to all participants so they can review the study guide outline and go through the escape room individually if desired. Participants were also encouraged to take group photographs/screenshots after successfully “escaping” as you do for a real Escape Room.

### Pearls

Learning points of this didactic activity are to review a high yield gastrointestinal/abdominal information. All participants should receive a link to a shared Google Document that is a comprehensive GI study outline compiled from Dr. Carol Rivers' Written Board Review resources via Ohio ACEP app, “The Ultimate Emergency Medicine Guide: The only EM book you need to succeed,” by Dr. Sajid Khan, Rosh Review Question Bank, and Hippo EM Board Review Videos.

## SMALL GROUP MATERIALS

### Appendix A Clue Answers and Master Sheet

#### Instructions

1. Provide link for escape room, bit.ly/giescaperoom (case sensitive, all lower case) to medical students/residents
2. Medical students/residents need to search the escape room for clues to click, which are links embedded into the escape room as highlighted below.
  [Fig f8-jetem-6-2-sg8]
3. “Click here to start” provides instructions for the escape room and should be clicked first. The rest of the clues can be clicked and completed in any order. The final clue, which is linked to the phone in the bottom left of the escape room scene, will need information gathered from correctly answering the other clues to complete.
4. Each clue that is clicked will open a new tab.
5. After the clues are solved, students/residents will be given letters they need to keep to solve the final clue to successfully escape.
6. After a clue is solved and letters are obtained, that tab can be closed and participants go back to tab with the escape room.
7. This escape room should be done on laptop/desktop for ease. It can be done on a tablet or phone, but each clue clicked will open another web browser page making it slightly more difficult.

#### Answers to clues

##### Clue #1

- Click: hematemesis from bitmoji cartoon
  ○ MCC (Major complications and comorbid conditions), UGIB (upper gastrointestinal bleeding): PUD, peptic ulcer disease
  ○ Other Causes UGIB: esophageal varices; esophagitis; Mallory Weiss tear; Dieulafoy lesion; Osler-Weber-Rendu Syndrome; Aorto-enteric fistula
  ○ Decrease all-cause mortality in esophageal variceal bleed: antibiotics
- Letters provided for successfully solving clue: **“GLA”**

##### Clue #2

- Click: IVF pole on right of screen
  ○ EHEC, (Enterohemorrhagic Escherichia coli) (O157:H7) → shiga toxin-producing; assoc. with Hemolytic Uremic Syndrome (HUS)
  ○ Salmonella → petting turtles; typhoid fever; relative bradycardia despite high fever
  ○ Shigella → febrile seizures in kids; Reiter’s syndrome
  ○ Vibrio vulnificus → raw shellfish/oysters; assoc. findings of skin bulla
  ○ Campylobacter jejuni → assoc. with Guillain Barre; can cause seizures; most common cause bacterial diarrhea
  ○ Staph aureus → picnic potato salad; heat-stable enterotoxin; most common foodborne illness
  ○ Botulism → home canned food; honey; diplopia; ptosis; descending paralysis; floppy baby syndrome
  ○ Vibrio cholera → “rice water diarrhea” = large volume loss
  ○ Listeria → deli meats, soft cheeses; increased risk of preterm labor
  ○ Clostridium difficile → aka Pseudomembranous colitis; diagnosis with stool toxin A & B immunoassay; complication = toxic megacolon
  ○ Scombroid → tuna or mahi mahi/dolphin fish, peppery taste; histamine-like reaction; treat with H1 + H2 blockers
  ○ Ciguatera → reef fish (grouper, barracuda, red snapper); acts on Na+ channels = Hot/Cold Reversal
  ○ Entamoeba histolytica → parasitic infection assoc. with liver abscess
  ○ Giardia lamblia → mountain streams/beavers; steatorrhea; diagnosis with stool antigen test
  ○ Cryptosporidium → most common cause chronic diarrhea in AIDS patients (pts)
  ○ Necator americanus → aka Hookworm; enters via bare feet from infected soil; affects lungs, GI
  ○ Enterobius vermicularis → aka Pinworm; pruritus ani at night = scotch tape test
- Letters provided for successfully solving clue: **“SGO”**

##### Clue #3

- Click: Code Cart
  ○ Hep B
    ■ Hep B Markers: Remember Ag = antigen, Ab = Antibody
      - HBsAg = HALLMARK OF active/acute DX = early active infection, positive in 1–10 weeks after exposure and is positive even before liver enzymes start to increase
      - HBsAb (aka Anti-HBs) = indicates IMMUNITY; HBsAb positive from vaccination or previous infection
      - HBcAb (aka Anti-HBc) = core antibody → can only be + from infection, never from just vaccination
        ○ HBcAb IgM = acute infection of Hep B
        ○ HBcAb IgM = chronic or recovery from Hep B
      - HBeAg = indicated HIGH INFECTIVITY
        [Table t2-jetem-6-2-sg8]
        **Table t2-jetem-6-2-sg8:**
        Hepatitis B					
        Clinical Status	HBsAg	Anti-HBs	Anti-HBc	HBeAg	Anti-HBe
        **Acute** hepatitis B infection	+	−	IgM	+	−
        **Chronic** hepatitis B with **active viral replication**	+	−	IgG	+	−
        **Chronic** hepatitis B with **low viral replication**	+	−	IgG	−	+
        **Recovery** from hepatitis B (immunity)	−	+	IgG	−	+ or −
        **Immunity** from **previous vaccination**	−	+	−	−	−
        (1) Remote infection and anti-HBs waned (2) Window period (3) Low level chronic infection	−	−	IgM	−	−
- Letters provided for successfully solving clue: **“W-”**

##### Clue #4

- Click: Burn poster back left corner of room
  ○ Bleach = Alkali caustic ingestion = liquefactive necrosis = needs serial airway assessments and esophagogastroduodenoscopy (EGD) within 12 hrs
- Letters provided for successfully solving clue: **“BL”**

##### Clue #5

- Click: gray drawers on right of screen with IV start kits etc
  [Table t3-jetem-6-2-sg8]
  **Table t3-jetem-6-2-sg8:**
  Crossword Clue	Answer
  ________ Discriminant Function Score >32 is prognostic of severe disease and pts may benefit from glucocorticoid therapy	MADDREY
  ________'s Triad = (1) fever, (2) RUQ pain, (3) jaundice and is concerning for ascending cholangitis	CHARCOT
  ________’s Criteria for Pancreatitis at admission includes Age >55 yrs. old, WBC >16k, Glucose >200, AST >250, LDH >350 are prognostic indicators of inpatient mortality at admission	RANSON
  Bilious vomiting in a newborn is considered ________ with volvulus until proven otherwise and is a surgical emergency	MALROTATION
  ________'s Disease has transmural skip lesions resembling cobble stoning anywhere in the GI tract from mouth to rectum and has extra-intestinal manifestations of uveitis and calcium oxalate renal calculi	CROHN
  Most common cause of lower GI bleeding	DIVERTICULOSIS
  ________ Inguinal Hernia is medial to inferior epigastric vessels	DIRECT
  Most common cause of painful lower GI bleeding	FISSURE
- Letters provided for successfully solving clue: **“ATCH”**

##### Clue #6

- Click: on US machine
- Answers in order:
  ○ Boerhaave Syndrome
  ○ Coin in esophagus
  ○ Coin in trachea
  ○ Acute cholecystitis
  ○ Necrotizing enterocolitis
  ○ Pyloric stenosis
  ○ Intussusception
  ○ Duodenal atresia
  ○ Sigmoid volvulus
  ○ Cecal volvulus
  ○ Small bowel obstruction
  ○ Button battery foreign body
- Letters provided for successfully solving clue: **“FORD”**

#### Final clue

- Click: spectra link phone
  ○ “GLASGOW-BLATCHFORD” Score. (the letters go in order of clues - any letters given together in clue stick together)
    ■ GLA from Clue #1
    ■ SGO from Clue #2
    ■ W- from Clue #3
    ■ BL from Clue #4
    ■ ATCH from Clue #5
    ■ FORD from Clue #6
  ○ Glasgow-Blatchford Score on MDCalc = 21 (use information from patient information sheet - available by clicking the vital signs on monitor)

### Appendix B GI Review Study Outline for Interactive Virtual Escape Room

This study guide was compiled from Dr. Carol Rivers' Written Board Review resources via Ohio ACEP app, “The Ultimate Emergency Medicine Guide: The only EM book you need to succeed” by Dr. Sajid Khan, RoshReview Question Bank, and Hippo EM Board Review Videos.

#### ESOPHAGUS

- Dysphagia:
  ○ Neuromuscular causes of dysphagia: CVA (Cerebrovascular accident) = Most Common Cause (MCC); polymyositis or dermatomyositis; scleroderma; myasthenia gravis (reversible w edrophonium); Multiple sclerosis, Amyotrophic lateral sclerosis, Parkinson’s; lead poisoning
  ○ Infectious causes of dysphagia: pharyngitis (Strep, Candida, herpes); abscess; polio; diphtheria; botulism; rabies; tetanus
  ○ Structural: esophageal webs (remember association w Plummer-Vinson syndrome; from chronic Gastroesophageal reflux disease (GERD); Schatzki Rings); achlasia; Zenker’s diverticulum; cancer
    ■ Schatzki’s Ring
      - Frequently found w/food boluses via EGD (esophagogastroduodenoscopy); Assoc. w/hiatal hernia
    ■ Achalasia
      - Symptoms (Sx): dysphagia to BOTH solids AND liquids b/c it’s a motility disorder
      - Physiology: Lower esophageal sphincter (LES) will not relax; Auerbach and Meissner plexus innervation are altered
      - Diagnosis (Dx): barium swallow study shows BIRD BEAK
    ■ Zenker’s Diverticulum: halitosis from food being trapped in outpouching
- Esophagitis
  ○ Caused by GERD; infectious (especially in immunocompromised) - Candida albicans, herpes simplex, varicella zoster, cytomegalovirus (CMV), mycobacterium); radiation; corrosive agents (alkali or acid ingestion); pill esophagitis
    ■ Caustic Ingestions
      - a**C**id ingestion ⇒ **C**oagulation ne**C**rosis thromboses the underlying blood vessels and forms a protective es**C**har/**C**oating
        ○ Do not induce vomiting, and typically do not give neutralizing agent
        ○ Specific scenario to give a neutralizing agent:
          ■ When HYDROFLOURIC ACID is ingested, give MAGNESIUM CITRATE to neutralize
      - a**L**kali ingestion ⇒ **L**iquefactive necrosis; ex. of a**L**kali = b**L**each
        ○ WORSE than acid ingestion; Typically need repeat airway assessment and EGD within 12 hrs
    ■ Pill esophagitis
      - Antibiotics (doxycycline, tetracycline); bisphosphonates; anti-inflammatory agents; potassium chloride; iron sulfate
  ○ Treatment (Tx) esophagitis: fluconazole for candida; assume herpes simplex in immunocompromised and give acyclovir
- Iatrogenic perforation = most common cause (MCC) esophageal perforation
- MALLORY WEISS TEAR
  ○ PARTIAL thickness tear at GASTRO-ESOPHAGEAL JUNCTION
  ○ Risk factors = alcoholism, hiatal hernia, gastritis/esophagitis
  ○ Sx: upper GI bleeding, dysphagia, odynophagia
  ○ Dx: EGD
- BOERHAAVE’S SYNDROME
  ○ FULL THICKNESS perforation of esophagus
  ○ Commonly left POSTEROLATERAL wall of distal esophagus
  ○ Sx: post-emetic chest pain/hypotension
  ○ Pathophys: forceful vomiting after drinking; iatrogenic
  ○ Dx:
    ■ Physical exam: HAMMAN’S CRUNCH = mediastinal air crunching heard w/heart beat
    ■ Chest radiograph (CXR):
      - Subcutaneous air
      - Left pneumothorax
      - Pneumomediastinum, wide mediastinum
        ○ Tubular artery sign = air tracking up towards neck
      - Pleural effusion
    ■ Confirm dx w UPPER GI STUDY with WATER SOLUBLE CONTRAST
  ○ Tx: IVF, IV abx (antibiotics--these pts can get sick!); GI vs surgery consult for EMERGENT EGD
    ■ morbidity/mortality due to shock and septicemia occur quickly (<48 hrs)
      [Fig f9-jetem-6-2-sg8]
- Esophageal Foreign Bodies:
  ○ Most common location in PEDIATRICS (PEDS) age <4 yrs.: C6 at cricopharyngeal muscle
  ○ Most common location in adults: T10 at lower esophageal sphincter (LES)
    ■ Sx: stridor, drooling, choking, vomiting, gagging
    ■ Dx: neck and chest radiography (or computed tomography (CT) for radiolucent foreign bodies-fish bones, plastic objects, tooth picks)
      - ***Remember kids like to swallow coins!
        ○ Sagittal orientation = coin in trachea → coin has to go through slot in between vocal cords; that is why oriented this way
        ○ Transverse orientation = coin in esophagus
          [Fig f10-jetem-6-2-sg8]
    ■ Tx: EGD for symptomatic (drooling or stridor) OR EGD for objects >2cm wide, >5cm long, irregular or sharp edges; otherwise, could try glucagon 1–2mg IV (or sublingual nitro or nifedipine) → relaxes the LES or try carbonated beverage, then monitor for passing of object w repeat CXR
      - ***BUTTON BATTERIES (halo or double rim/ring around edge of coin shape) NEED TO BE REMOVED IMMEDIATELY b/c can cause esophageal erosion/perforation within 4–6 hrs of ingestion
      - Single magnet ingestion can be managed expectantly; multiple magnets need to be removed
      - Ileocecal valve = most common site of perforation
- PEPTIC ULCER DISEASE (PUD) = MOST COMMON CAUSE UPPER GI BLEED (MCC UGIB even in pts with varices!!)
  ○ Biggest risk factor for PUD = H. PYLORI; NSAID, smoking, stress, prolonged use of corticosteroids, caffeinated beverages = other risk factors
  ○ Sxs:
    ■ GASTRIC peptic ulcer = pain after eating, pain gets WORSE WITH FOOD = WEIGHT LOSS; can turn into gastric CANCER so should have biopsy performed with EGD
    ■ DUODENAL peptic ulcer = pain 2–3 hrs after eating, pain gets BETTER with food ingestion = no associated weight loss; more likely to BLEED
  ○ Dx: EGD with biopsy to assess malignant vs benign peptic ulcers
    ■ H. pylori dx via serology or urea breath tests or rapid urea/C10 test or histology from EGD or IgG H pylori serum test
  ○ Tx:
    ■ Tx PUD:
      - Stop causal irritant (NSAID, smoking, alcohol)
      - Antacids for pain relief and to accelerate healing by neutralizing gastric acid
      - H2 blockers (inhibit gastric acid secretion)
      - PPI (protein pump inhibiters block acid secretion by inhibiting the H+/K+ ATPase pump of parietal cells)
      - Sucralfate (aka Carafate = surface protectants that bind to ulcer lesion to prevent further damage from acid by absorbing bile acids, inhibiting pepsin activity, increasing mucosal prostaglandin production)
      - Bismuth compounds (diminish pepsin activity, increase mucus secretion, create a barrier to ulcerated surfaces, augment prostaglandin synthesis, bactericidal effect on H. pylori (used in quadruple therapy)
      - EGD for significant UGIB from PUD
        ○ Remember CTA only helpful/able to pick up bleed location if rate of bleeding is >0.5 mL/min
    ■ Tx PUD from NSAID = Misoprostol (aka Cytotec aka synthetic prostaglandin E1 analogue)
      - **Misoprostol CONTRAINDICATED in pregnant women or women of child bearing age not using contraceptive measures because misoprostol can cause spontaneous abortion
    ■ Tx H. PYLORI
      - Triple Therapy: ^(1)^ omeprazole, ^(2)^ amoxicillin (substitute metronidazole for allergic pts), ^(3)^ clarithromycin
      - Quad Therapy: ^(1)^ bismuth subsalicylate (Pepto-Bismol), ^(2)^metronidazole, ^(3)^tetracycline, and ^(4)^ PPI or ranitidine
  ○ Complications of PUD: UGIB, perforation
- Upper GI Bleed:
  ○ Sx: hematemesis, melena, nasogastric lavage (NG) with blood or coffee ground
    ■ NG does NOT decrease mortality or transfusion requirements; clear NG aspirate may miss up to 15% clinically relevant lesions
  ○ Causes of UGIB:
    ■ 1. PUD!!!!!! Most common cause UGIB
    ■ 2. ESOPHAGEAL VARICES
      - Esophageal varices Tx =
        ^(1)^ PPI
        ^(2)^ somatostatin (aka octreotide = splanchnic vasoconstriction) - does not decrease mortality but does increase initial hemostasis and decrease rebleeding
        ^(3)^ vasopressin
        ^(4)^ abx = rocephin/ceftriaxone OR ciprofloxacin (b/c cirrhosis pts. are immunocompromised and need abx prophylaxis)
          ■ **in pts. w cirrhosis and UGIB prophylactic abx decreases all-cause mortality, rebleeding, death from bacterial infection, and length of hospital stay**
        ○ EGD = definitive tx for esophageal varices with band ligation or sclerotherapy
          ■ To temporize can use Sengstaken-Blakemore tube until EGD can performed if needed
          ■ If EGD unsuccessful, perform angiography with embolization of gastric vein
        ○ Patients might need TIPS procedure (**T**ransjugular **I**ntrahepatic **P**ortosystemic **S**hunt) for refractory bleeding
    ■ 3. Esophagitis
    ■ 4. Mallory Weiss (PARTIAL thickness) tear
    ■ 5. Dieulafoy lesion (torturous arteriole in stomach that erodes and bleeds)
    ■ 6. Osler-Weber-Rendu Syndrome (aka hereditary hemorrhagic telangiectasia) - autosomal dominant inheritance; frequently presents with epistaxis; telangiectasis of skin/mucous membranes/GI tract causing recurrent GI bleeding
    ■ 7. Aorto-enteric fistula
  ○ Fun facts about UGIB:
    ■ Black stools - false negatives (aka heme occult negative) cause by Pepto-Bismol and iron tablets
    ■ Increased BUN:creat ratio >30
    ■ PPI for undifferentiated UGIB does NOT improve mortality; it may decrease re-bleeding
      - Comparing PPI to H2 blockers, PPI have more benefit when compared to H2 blockers for tx of UGIB (lower rate of re-bleeding, shorter hospital stays, lower rates of transfusion)
    ■ Glasgow-Blatchford Score - predicts urgent/emergent need for intervention in UGIB
      - Hemoglobin (hgb), BUN, systolic blood pressure, sex, heart rate >100, melena, syncope, hx hepatic disease, cardiac failure
  ○ Tx of UGIB:
    ■ 2 large bore (at least 18 gauge) IV; crystalloid IVF infusion (2L bolus, continuous until vital signs stabilize or 40mL/kg have been given to adult)
    ■ Cardiac monitor, O2 as needed
    ■ Labs, immediate transfusion of type-specific/cross-matched blood if available or emergent transfusion with Type O negative blood if needed
    ■ Consider reversal of coagulation abnormalities (vit K, fresh frozen plasma (FFP), prothrombin complex concentrate (PCC), activated recombinant factor VII, or specific novel anticoagulants reversal agents)
    ■ ***Restrictive transfusion strategy (only transfuse if hgb less than 7g/dL) reduces mortality in UGIB***
      [Fig f11-jetem-6-2-sg8]
- Causes of Lower GI Bleed (LGIB) (hematochezia)
  ○ 1. DIVERTICULOSIS!!!!!! Most common cause (PAINLESS) LGIB
  ○ 2. Anal fissures
  ○ 3. Hemorrhoids
  ○ 4. Colon mass/polyps
  ○ 5. Ulcerative colitis
  ○ 6. Diverticulitis
  ○ 7. Arteriovenous malformation
  ○ 8. Aorto-enteric fistula

#### LIVER

- Hepatitis (Hep)- **A**L**T is more specific for **L**iver injury!
  ○ Hep A
    ■ “vowels hit your bowels = Hep A & Hep E spread by fecal-oral”; RNA virus
    ■ Anti-HAV IgM=acute infection; Anti-HAV IgG=past infection or vaccination
    ■ Vaccine available - given at least 2 weeks before travel to endemic areas
    ■ If patient presents within 2 weeks of exposure to hep A, give immune globulin (aka gamma globulin or serum immune globulin)!
  ○ Hep B
    ■ Transmitted percutaneous, parental, or sexual exposure; DNA virus
    ■ Small chance turning into chronic hepatitis
    ■ Co-infection of Hep B and Hep D = most common cause of fulminant hepatic failure
    ■ ******* HEP B MARKERS!!!!!! Remember Ag = antigen, Ab = Antibody**
      - **HBsAg = HALLMARK OF active/acute DX = early active infection, positive in 1–10 weeks after exposure and is positive even before liver enzymes start to increase**
      - **HBsAb (aka Anti-HBs) = indicates IMMUNITY; HBsAb positive from vaccination or previous infection**
      - **HBcAb (aka Anti-HBc) = core antibody → can only be + from infection, never from just vaccination**
        ○ **HBcAb IgM = acute infection of Hep B**
        ○ **HBcAb IgM = chronic or recovery from Hep B**
      - **HBeAg = indicated HIGH INFECTIVITY**
    ■ Hep B exposure Tx:
      - Hep B exposure in unvaccinated pts tx = give hep B immune globulin (HBIG) + vaccine
      - Hep B exposure in vaccinated pts tx = Check HBsAb levels
        ○ If HBsAb normal (>10), no treatment needed
        ○ If HBsAb titer LOW, give immune globulin + vaccine BOOSTER
          [Table t4-jetem-6-2-sg8]
          **Table t4-jetem-6-2-sg8:**
          Hepatitis B					
          Clinical Status	HBsAg	Anti-HBs	Anti-HBc	HBeAg	Anti-HBe
          **Acute** hepatitis B infection	+	−	IgM	+	−
          **Chronic** hepatitis B with **active viral replication**	+	−	IgG	+	−
          **Chronic** hepatitis B with **low viral replication**	+	−	IgG	−	+
          **Recovery** from hepatitis B (immunity)	−	+	IgG	−	+ or −
          **Immunity** from **previous vaccination**	−	+	−	−	−
          (1) Remote infection and anti-HBs waned (2) Window period (3) Low level chronic infection	−	−	IgM	−	−
  ○ Hep C
    ■ Acquired via blood transfusions, intravenous drug abuse (IVDA)
    ■ 50% will develop chronic infection → cirrhosis and hepatocellular carcinoma
    ■ Healthcare workers who sustain needle stick injury from known Hep C + patient should be tested immediately and every 2 months x3 (ending at 6 months after exposure)
      - Chance of getting Hep C after needle stick from HCV positive source ~1.8%
    ■ Prophylaxis not available, curative therapy is available
  ○ Hep D
    ■ Co-infection with Hep B
  ○ Hep E
    ■ “vowels hit your bowels = Hep A & Hep E spread by fecal-oral”; RNA virus
    ■ Fulminant liver failure particularly in pregnant pts
- Toxic Hepatitis Causes:
  ○ Tylenol → saturation of the sulfate and glucuronide pathways and depletion of glutathione ⇒ buildup of toxic metabolite NAPQI
  ○ Mushroom → Amanita phalloides “death cap”
  ○ Halothane - postop fever, rash, eosinophilia
  ○ Methyldopa - rash, arthralgias, lymphadenopathy then jaundice
  ○ Isoniazid (INH) - worse in alcoholic or concurrent use of rifampin or pyrazinamide
  ○ Phenytoin
  ○ Anabolic steroids, oral contraceptives, chlorpromazine, haloperidol, verapamil, phenobarbital
  ○ alcohol
- Alcoholic Hepatitis = (aspartate aminotransferase) AST: alanine aminotransferase (ALT) is 2:1 (Let’s toAST our 2 beers!)
  ○ Presence of encephalopathy = strongest short-term mortality predictor
  ○ MADDREY DISCRIMINANT FUNCTION SCORE >32 is prognostic of severe disease and pts may benefit from glucocorticoid therapy
  ○ Alcoholic Cirrhosis (aka Laennec Cirrhosis)
    ■ Sxs: jaundice, spider hemangioma, palmar erythema, gynecomastia, muscle wasting, Dupuytren’s contracture, ascites, pedal edema
    ■ Labs: macrocytic anemia, leukopenia, thrombocytopenia, prolonged INR >1.5, hypOalbumin, hypOnatremia, hypOkalemia
  ○ Esophageal Varices Bleed: Tx = ^(1)^ PPI; ^(2)^ somatostatin (aka octreotide = splanchnic vasoconstriction); ^(3)^ vasopressin; ^(4)^ abx = rocephin/ceftriazone OR ciprofloxacin (b/c cirrhosis pts are immunocompromised and need abx prophylaxis)
    ■ **in pts w/cirrhosis and UGIB, prophylactic abx decrease all-cause mortality, rebleeding, death from bacterial infection, and length of hospital stay**
    ■ EGD = definitive tx for esophageal varices with band ligation or sclerotherapy
      - To temporize can use Sengstaken-Blakemore tube until EGD can be performed if needed
      - If EGD unsuccessful, perform angiography with embolization of gastric vein
      - Patients might need TIPS procedure (**T**ransjugular **I**ntrahepatic **P**ortosystemic **S**hunt) for refractory bleeding
  ○ Hepatic Encephalopathy
    ■ Sxs: altered mental status, confusion, coma, asterixis, sleep inversion (sleep during day/awake at night) is early sign
    ■ Pathophys:
      - Azotemia = MCC → azotemia caused by GI bleed, Infection
      - Electrolyte abnormalities
      - Medication nonadherence
    ■ Serum ammonia is normally elevated but level does not correlate w/sx
    ■ Tx: lactulose to eliminate ammonia, decrease dietary protein, consider empiric abx (rifaximin or neomycin)
  ○ Hepatorenal Syndrome (HRS)
    ■ Pathophys: vasoconstriction and shunting of blood away from renal cortex → decreased GFR → decreased renal output → azotemia
    ■ Prognosis is dismal with nearly 100% mortality
    ■ Type 1 HRS (more serious): twofold increase in creatinine to a level greater than 2.5 in less than 2 weeks; might have urine output less than 500 cc/day
    ■ Type 2 HRS: hallmark = ascites refractory to diuretic use; less severe renal impairment than type 1 (any renal impairment that does not meet Type 1 criteria)
    ■ MELD SCORE = stratifies severity of end stage liver disease for transplant planning
  ○ Spontaneous Bacterial Peritonitis (SBP)
    ■ Sxs: in pts with ascites, abdominal pain and fever (and consider in GI bleed pts!)
    ■ Dx: ascitic fluid with absolute neutrophil count/aka polymorphonuclear leukocytes (PMN) >250 or WBC >1,000 or pH <7.35 or positive ascitic culture
    ■ Most common organism = E.coli
    ■ Tx: CEFOTAXIME or CEFTRIAXONE or CEFEPIME

#### GALLBLADDER

- Cholelithiasis
  ○ Risk Factors: F’s → Female (#1 RF), Fat, Fertile, Forty, Fair skin
  ○ Dx: Ultrasound (US)
  ○ Tx:
    ■ Pain control - GYCOPYRROLATE (anticholinergic to decrease spasm); NSAIDs (IV Toradol); opiates as needed
    ■ Cholecystectomy
- Cholecystitis:
  ○ Pathophys: obstruction of cystic duct
  ○ Dx: US: gallstones or sludge, pericholecystic fluid, GB wall thickening >3mm, common bile duct >6mm, sonographic Murphy’s sign
    ■ Remember normal common bile duct (CBD) is 5mm (+1mm for every decade older than 50 yrs. old)
  ○ If US negative but suspicion high, HIDA SCAN (nuclear scintigraphy) has HIGHEST SENSITIVITY & SPECIFICITY for cholecystitis
  ○ Calculous cholecystitis = MCC acute pancreatitis (gallstone goes into CBD and occludes pancreatic duct at sphincter of Oddi)
  ○ Rare variants of cholecystitis with high mortality rates:
    ■ EMPHYSEMATOUS CHOLECYSTITIS
      - air in GB wall; Risk factors - MEN and DIABETES; caused by Clostridium perfringens, E. coli, B. fragilis, Klebsiella; SURGICAL EMERGENCY due to risk of GB gangrene and perforation
    ■ ACALCULOUS CHOLECYSTITIS
      - US shows same US findings (thickened GB wall, perichole fluid) but no gallstones
      - These patients are acutely ill - more common in elderly, ICU, burn pts/major trauma, immunocompromised (AIDS, diabetes mellitus) pts
      - High mortality from gangrene or perforation
- CHOLEDOCHOLITHIASIS = gallstones in CBD
  ○ Sxs: severe, pain, nausea, biliary colic, pancreatitis to cholangitis
    ■ ASCENDING CHOLANGITIS = obstruction of CBD leads to ascending bacterial (E. coli) infection
      - CHARCOT’S TRIAD = (1) fever, (2) RUQ pain, (3) jaundice
      - REYNOLD’S PENTAD = Charcot’s triad + (4) confusion, (5) hypotension
    ■ Tx: Surgical emergency - endoscopic retrograde cholangiopancreatography (ERCP) or surgery plus abx (piperacillin/tazobactam)
      [Fig f12-jetem-6-2-sg8]
- Abnormal Bilirubin
  ○ Indirect/unconjugated bilirubin: Elevated from increased hemolysis or elevated when there is hepatocellular injury and decreased conjugation
  ○ Direct/conjugated bilirubin: Increased due to obstruction of secretion via stool
  ○ PEDIATRIC JAUNDICE
    ■ PATHOLOGIC Pediatric Jaundice
      - Jaundice in first 24 hrs of life is PATHOLOGIC → could progress to kernicterus; Jaundice persisting more than 2 weeks of life
      - conjugated/direct hyperbilirubinemia = pathologic
    ■ Physiologic Pediatric Jaundice
      - Develops within first week and resolves by age 2 weeks

#### PANCREAS

- PANCREATITIS
  ○ Causes of Pancreatitis = **GET SMASHED** → **G**allstones; **E**tOH; **T**rauma; **S**teroids; **M**umps (or other infections like infectious mono, mycoplasma, coxsackie, adenovirus, salmonella, viral hepatitis) or **M**om-to-be (pregnant); **A**utoimmune; **S**corpion bites; **H**yperlipidemia/**H**ypercalcemia; **E**RCP; **D**rugs (thiazides, furosemide, estrogen, salicylates, propofol, sulfa drugs)
  ○ Lipase higher sensitivity than amylase; ALT test has low sensitivity but high specificity for biliary etiology in cases of acute pancreatitis
  ○ Signs/PE findings of Pancreatitis:
    ■ CULLEN’S Sign = umbilical ecchymosis from hemoperitoneum
    ■ GREY-TURNER Sign = flank ecchymosis from retroperitoneal bleed
  ○ RANSON’S CRITERIA FOR PANCREATITIS = prognostic indicators of inpatient mortality for acute pancreatitis
    ■ RANSON’S CRITERIA FOR PANCREATITIS @ ADMISSION
      - (1) AGE > 55 YRS OLD
      - (2) WBC > 16k
      - (3) GLUCOSE > 200
      - (4) AST > 250
      - (5) LDH > 350
    ■ RANSON’S CRITERIA FOR PANCREATITIS @ 48 HRS
      - (1) Calcium <8
      - (2) Hct decreased by 10%+ (suggests hemorrhagic pancreatitis)
      - (3) PaO2 <60 (suggests acute respiratory distress syndrome [ARDS])
      - (4) BUN increased by 5+
      - (5) Base deficit >4
      - (6) sequestration of fluids
  ○ Tx: supportive care (IVF, NPO, pain meds)
  ○ Complications to know about → pancreatic pseudocyst/phlegmon; pancreatic abscess; hyperglycemia; hypocalcemia; volume loss → 3rd spacing → acute tubular necrosis renal failure; pleural effusions (L>R); ARDS; disseminated intravascular coagulation (DIC)
    [Fig f13-jetem-6-2-sg8]

#### SPLEEN

- Asplenic pts (sickle cell, trauma) at risk for encapsulated organism infection (Strep pneumo; H. influenza; Neisseria meningitidis) → cover these patients w/Ceftriaxone AND Vancomycin
- Portal Hypertension (HTN) = MCC large spleen
- Epstein-Barr virus (EBV) infection/MONO = sore throat atypical lymphocytes, morbilliform rash after Penicillin; EBV infection/MONO causes enlarged spleen = NO CONTACT SPORTS for at least 3 weeks!!!!!

#### PEDIATRIC GI

- MALROTATION with volvulus
  ○ Age 3–7 DAYS old
  ○ Sxs: BILIOUS VOMITING = SURGICAL EMERGENCY IN NEWBORN
  ○ Pathophys: small bowel twists around the superior mesenteric artery (SMA) resulting in vascular compromise ⇒ ischemia and bowel necrosis - sepsis and fluid losses follow
  ○ Dx: upper GI series
  ○ Tx: SURGERY
- Omphalitis
  ○ Superficial polymicrobial cellulitis of umbilical cord, could progress to necrotizing fasciitis so you need to consult pediatric surgeon early and start IV abx
- Necrotizing Enterocolitis (NEC)
  ○ PREMATURE infants
  ○ Sxs: poor feeding, vomiting, abdominal (abd) distention, guaiac positive stools, blue appearing abdomen
    ■ Remember, swallowed maternal blood can cause false impression of neonate with bloody stools -- use the Apt test to make the distinction → apt test = enzymatic solution added to neonate’s stool, will turn brown if swallowed maternal blood is present OR will remain red if blood is fetal intestine blood
  ○ Dx: X-ray shows PNEUMATOSIS INTESTINALIS (air in bowel walls)
  ○ Tx: bowel rest, nasogastric (NG) tube, abx; possibly need surgery
- PYLORIC STENOSIS
  ○ NON-BILIOUS PROJECTILE VOMITING up to 3 months of age
  ○ OLIVE-SHAPED PALPABLE MASS IN RUQ
  ○ PYLORIC STENOSIS = HYPOKALEMIC HYPOCHLOREMIC METABOLIC ALKALOSIS
  ○ Dx: US measures pylorus thicker than 4mm or longer than 15mm
- INTUSSUSCEPTION typically occurs in AGES 3–36 MONTHS
  ○ RUQ INTERMITTENT abd pain = colicky, draws legs up into belly
  ○ SAUSAGE SHAPED PALPABLE ABD MASS
  ○ Intussusception is most common cause of bowel obstruction in kids <2 yrs. and is the most common cause of abdominal emergency in kids!
  ○ Intussusception is associated with viral syndromes (hypertrophied lymphoid tissue develops in bowel and acts as a lead point); Meckel’s diverticulum; Henoch-Schonlein Pupura (HSP); cystic fibrosis (thick stool)
  ○ Late finding of INTUSSUSCEPTION = CURRANT JELLY STOOL
  ○ Dx: US = target sign
  ○ AIR or BARIUM/CONTRAST ENEMA = dx and tx of intussusception
    [Fig f14-jetem-6-2-sg8]
- Meckel’s Diverticulum
  ○ Sxs: PAINLESS RECTAL BLEEDING
  ○ Rule of 2’s: present in 2% of population; only 2% ever develop sxs; located within 2 ft proximal to ileocecal valve; 2cm long and 2cm wide; half of all pts develop sxs by age 2
- Duodenal Atresia
  ○ Congenital obstruction of duodenum; associated w/Down’s Syndrome and pregnancies complicated by polyhydramnios
  ○ Dx: “Double-Bubble” sign on X-ray
  ○ Tx: surgery
- Hirschsprung's Disease aka “Congenital Aganglionic Megacolon”
  ○ Absence of ganglion cells in distal colon/rectosigmoid area
  ○ Sx: delayed passage of meconium, absence of stool in rectal vault, abd distension, bilious emesis
    ■ Newborn sxs = failure to pass meconium
    ■ Pediatric sxs = chronic constipation
  ○ Dx: rectal biopsy or rectal manometry
  ○ Complications = toxic megacolon!
- Hemolytic-Uremic Syndrome (HUS)
  ○ Caused by: enterohemorrhagic E. coli (O157:H7)
  ○ Sxs: watery → bloody diarrhea; periorbital edema
  ○ HUS TRIAD
    ■ (1) acute renal failure
    ■ (2) thrombocytopenia
    ■ (3) Microangiopathic Hemolytic Anemia (see schistocytes on smear)

#### BOWELS

- Ileus
  ○ Causes of ileus = POST-OPERATIVE, drugs (opioids or anticholinergics), electrolyte abnormalities (hypOcalcemia or hypOkalemia); hypOthyroidism
  ○ Sx: hypOactive bowel sounds; multiple dilated fluid filled loops of bowels
  ○ Tx: bowel rest (NPO), IVF, consider NG tube
- Bowel Obstruction = air- fluid levels on imaging
  ○ Causes of small bowel obstruction (SBO) - MCC is ADHESIONS; 2nd MCC SBO = hernia; other causes = neoplasms, intussusception, gallstone ileus, Crohn’s, foreign bodies
  ○ Large bowel obstruction = MASS/MALIGNANCY is MCC large bowel obstruction; 2nd MCC is diverticulitis; other causes = volvulus, fecal impaction
  ○ Sxs: hypERactive bowel sounds; cessation of flatus/bowel movements; pain, vomiting, distention, +tympany to percussion
  ○ Dx: X-ray → demonstrates air-fluid levels; CT to identify transition point
  ○ Tx: IVF, correction of electrolytes, NG tube, early surgical consultation, possible abx coverage
- OGILVIE SYNDROME = acute colon pseudo-obstruction
  ○ Found mostly in elderly, bedridden pts; caused by autonomic dysfunction (NO mechanical obstruction) leading to massive dilation of the colon (>10cm)
  ○ Tx = colonic decompression and neostigmine
- CECAL VOLVULUS
  ○ RLQ pain in younger patients (age 20s–30s most commonly) → Risk factors = marathon runners, 3rd trimester pregnancy
  ○ XR findings = Bird Beak Sign or kidney or “coffee bean”
  ○ CT findings = Whirl/Hurricane sign
  ○ Tx: early and immediate surgical reduction as decompression alone will not reduce b/c it is a closed-loop obstruction
- SIGMOID VOLVULUS - more common than cecal volvulus
  ○ Risk factors = elderly, bed bound, psychiatric/anticholinergic medications
  ○ Sxs: triad of pain, distention, & obstipation
  ○ Dx: X-ray shows “bent inner tube” shape with LLQ appearing empty
  ○ If not treated, sigmoid volvulus can lead to bowel necrosis, sepsis, death!
  ○ Tx: flexible sigmoidoscopy; rectal tube
    ■ ER tx - decompression w/NG and rectal tube via sigmoidoscope or barium enema; consultation w/GI or surgery, starting broad spectrum abx
- Inflammatory Bowel Disease - Crohn’s & Ulcerative colitis; peak incidence age 15–40
  [Table t5-jetem-6-2-sg8]
  **Table t5-jetem-6-2-sg8:**
  CROHN’S	ULCERATIVE COLITIS
  Any part of GI tract, commonly affects ileum (RLQ pain)	Affects rectum & colon
  SKIP LESIONS; cobblestone appearance	CONTINUOUS
  TRANSMURAL = affects ALL layers	SUBMUCOSAL/only affects superficial layer (muscle and serosa spared)
  More common in Jewish population; associated with nocturnal diarrhea and weight loss	Associated w significantly increased risk of colon cancer
  Associated w/perirectal fistulas/abscesses, non-midline fissures; extraintestinal manifestations = arthritic (ankylosing spondylitis); vascular (vasculitis); dermatologic (erythema nodosum, pyoderma gangrenosum); ophthalmic (UVEITIS, episcleritis, iritis, conjunctivitis); renal calculi (Associated with CALCIUM OXALATE CRYSTALS)	Complications include toxic megacolon (colonic distention >6cm, also from C. Diff); LGIB; perforation; obstruction from strictures; colon cancer
  Tx Crohn’s: steroids, mesalamine (5-ASA derivative); cipro or metronidazole; azathioprine and 6-mercaptopurine (immunosuppressants); anti-TNF (infliximab, adalimumab, certolizumab pegol)	Tx of ulcerative colitis: 5-ASA derivative such as mesalamine or olsalazine; corticosteroids; azathioprine, cyclosporine, 6-mercaptopurine
- Pseudomembranous enterocolitis = Clostridium Difficile (C. Diff)
  ○ Yellowish pseudomembranous-like plaques that overlie and replace necrotic intestinal mucosa particularly in rectosigmoid
  ○ Antibiotic ingestion alters gut flora ⇒ proliferation of C. Diff toxin-producing bacteria
    ■ Abx associated with causing C. Diff:
      - Fluoroquinolones (in particular strain NAP-1/027 of C. Diff), clindamycin, cephalosporins, and penicillins
  ○ Nosocomial transmission among hospitalized pts
  ○ Tx: oral vancomycin first line (old tx PO metronidazole)
  ○ Complication = toxic megacolon
- Irritable bowel syndrome (IBS) → ROME Criteria = recurrent abdominal pain or discomfort for at least 3 days per month in the last 3 months associated with at least 2 of the following:
  ○ Improvement with defecation
  ○ Onset associated with change in frequency of defecation
  ○ Onset associated with change in form/appearance of feces
    ■ IBS Tx: high fiber/bulk forming; antidiarrheals, antispasmodics
- Mesenteric Ischemia
  ○ Risk factors = vasculopathy (AFib, CAD, DM, HTN) or hemodialysis pts (low flow non-occlusion cause)
  ○ Sxs: PAIN OUT OF PROPORTION TO EXAM; guaiac positive or grossly bloody stool
  ○ Pathophys: arterial embolism (MCC); arterial thrombosis (pain after eating); venous thrombosis (hypercoagulable state such as polycythemia vera, antithrombin III deficiency); non-occlusive (hypoperfusion to mesenteric vasculature due to low cardiac output from hemodialysis; congestive heart failure (CHF); cardiogenic or septic shock; medications that cause splanchnic vasoconstriction such as digoxin, digitalis, or vasopressors)
  ○ Dx: lactate elevated late in course; CTA IS GOLD STANDARD DX
  ○ Tx: early vascular surgical consultation (possibly need to consult interventional radiology for angioplasty), aggressive IVF, broad spectrum abx, NPO, heparin, papaverine (decrease arterial vasospasm/dilate mesenteric arteries)
- Appendicitis
  ○ ***Appendicitis = MCC of emergent surgery; most common surgical emergency in pregnancy; most common surgical emergency in children
  ○ Sx: abdominal pain (classic periumbilical with migration to RLQ); anorexia; nausea/vomiting; fever; diarrhea
  ○ Physical Exam:
    ■ RLQ pain most sensitive finding (remember pregnant pts get appendicitis too and will be different location since gravid uterus displaces normal anatomy)
    ■ Tender to palpation over McBurney’s point
    ■ Rebound Tenderness
    ■ Rovsing sign = when you palpate LLQ and get RLQ pain
    ■ Psoas sign = pain w/extension R hip
    ■ Obturator sign = pain w/rotation of flexed right hip
    ■ Cervical motion tenderness
  ○ Dx:
    ■ CT: periappendiceal fat stranding = most specific finding; IV contrast and rectal contrast may increase sensitivity; dilated appendix >6mm on CT
    ■ US: graded compression on US; non-compressible, immobile appendix >6mm diameter on US
      - US is ~40% sensitive and 90% specific
      - Very poor sensitivity in appendiceal perforation
    ■ MRI in pregnant pts who US in inconclusive
    ■ Diagnostic laparoscopy
  ○ DDx of Appendicitis:
    ■ Mesenteric adenitis, Yersinia gastroenteritis, pelvic inflammatory disease, ectopic pregnancy, ovarian cyst, pyelonephritis, Crohn’s disease, diverticulitis
  ○ Tx: surg consult; IV abx (IV zosyn or IV cefoxitin or IV cefotetan or IV ampicillin, gentamicin, and metronidazole/clindamycin)
    ■ Several studies show nonsurgical management (treat w/abx only) of acute uncomplicated appendicitis is acceptable
      - NOTA study (Nonoperative Treatment for Acute Appendicitis) showed good outcomes w/2 yr. recurrence rate of only 14% after treatment with amoxicillin/clavulanic acid only
- Diverticulosis
  ○ REMEMBER DIVERTICULOSIS = MCC LGIB!
  ○ Diverticula = saclike herniations of colonic mucosa and submucosa through muscularis
  ○ Tx: self-limited, high fiber and stool softeners
- Diverticulitis
  ○ Sxs: crampy LLQ pain; nausea, bloating, constipation OR diarrhea; painless rectal bleeding
  ○ Tx for uncomplicated diverticulitis:
    ■ High fiber diet
    ■ Abx to cover E. coli and B. fragilis/anaerobes and gram negative:
      - Trimethoprim-sulfamethoxazole (TMP-SMX) + metronidazole OR
      - Ciprofloxacin + metronidazole OR
      - Amoxicillin/clavulanate for 10 days
  ○ Complicated diverticulitis = micro perforation, abscess, phlegmon, fistula
    ■ Tx for complicated diverticulitis: IV abx to cover aerobic and anaerobic
      - IV Ciprofloxacin + IV metronidazole OR
      - IV ticarcillin/clavulanate
      - IV ampicillin/sulbactam
      - IV Imipenem
    ■ Surgery consult for free air
- Hernias
  ○ Lifetime risk of developing groin hernia is 25% in men
  ○ 96% of groin hernias are INGUINAL!
    ■ Inguinal hernias
      - DIRECT Inguinal hernia = Hesselbach triangle, MEDIAL to inferior epigastric vessels
      - INDIRECT Inguinal hernia = most common type of hernia; protrudes LATERAL to inferior epigastric vessels through a congenital defect in processus vaginalis; frequently incarcerates especially in infancy
  ○ Dx Hernias - use US first!
    [Table t6-jetem-6-2-sg8]
    **Table t6-jetem-6-2-sg8:**
    Hernias			
    Type	Pathophysiology	Location	Characteristics
    **Direct inguinal**	Defect of the transversalis fascia in Hesselbach triangle	Groin, **medial** to the inferior epigastric vessels	Middle-aged or **elderly men**
    **Indirect inguinal**	Persistent processus	Groin, **lateral** to the inferior epigastric vessels	Congenital, trauma, can descend into **scrotum**
    **Femoral**	Through femoral canal	**Upper thigh**, medial to the femoral vein	Most common in **women**, risk of incarceration and strangulation
    **Incisional/ventral**	Breakdown of fascial closure from prior surgery	Site of **previous surgery**	Usually **asymptomatic**, increase in size with straining
    **Umbilical**	Through the fibromuscular umbilical ring	Umbilicus	Repair if persists beyond 5 years
    **Obturator**	Through the large obturator canal	Deep structures, not visualized externally	**Female** > male
    **Epigastric**	Through **defects in aponeurosis of rectus sheath**	Midline between umbilicus and xiphoid process	Middle-aged and young children
- DIARRHEA
  ○ MCC Diarrhea = VIRAL
    ■ Norovirus = MCC viral adult diarrhea; associated w/cruise ships
    ■ Rotavirus = MCC viral pediatric diarrhea; associated w/winter season
      - Vaccine given to babies at 2, 4, & 6 months of age
- **Invasive BLOODY Bacterial Diarrhea**
  ○ Dx: will typically have fecal blood and fecal leukocytes
  ○ TX: IVF, electrolytes replacement; no benefit of abx in healthy immunocompetent pts; those w severe diarrhea, high fever, or who need hospitalization can benefit from 3–7 days of abx cipro or Bactrim or amoxicillin
    [Table t7-jetem-6-2-sg8]
    **Table t7-jetem-6-2-sg8:**
    Bacteria	Source	Other “BizzBuzz”
    Enteroinvasive/hemorrhagic E. coli (EHEC; O157:H7)	Undercooked hamburger; petting zoos	- Shiga toxin-producing - NO abx as increases risk of Hemolytic Uremic Syndrome (HUS) → HUS triad: (1) acute renal failure; (2) thrombocytopenia; (3) Microangiopathic Hemolytic Anemia (see schistocytes on smear)
    Campylobacter jejuni	Raw chicken; unpasteurized milk	- MCC bacterial diarrhea - Pseudo appendicitis - Associated w/Guillain Barre, Hemolytic uremic syndrome (HUS), Reiter syndrome (nongonococcal urethritis, polyarthritis, conjunctivitis) - Chance of seizures - Tx: azithromycin
    Salmonella	Raw eggs; poultry; touching turtles	- Typhoid Fever - Rose spots rash = “salmon patches” - High risk pts: kids <5 yrs., immunocompromised, splenectomy pts, hemolytic anemias including sickle cell anemia - ***RELATIVE BRADYCARDIA (bradycardia despite high fever)*** - Tx: cipro or Levaquin or Bactrim
    Shigella	Contaminated water	- Seizures in kids <2 yrs. old - Tx: cipro or azithromycin or IV rocephin - Other complications besides febrile seizures: arthralgias, Reiter syndrome (nongonococcal urethritis, polyarthritis, conjunctivitis), HUS
    Vibrio parahaemolyticus	Raw shellfish	- Supportive Tx, cipro or Bactrim if severe
    Vibrio vulnificans	Raw shellfish/oysters	- Skin findings of bulla
    Yersinia enterocolitica	undercooked tofu or pork	- Pseudo appendicitis - Post-infectious sequelae include erythema nodosum and reactive polyarthritis
- **Non-invasive WATERY Bacterial Diarrhea**
  [Table t8-jetem-6-2-sg8]
  **Table t8-jetem-6-2-sg8:**
  Bacteria	Source	Other “BizzBuzz”
  Staph aureus	Picnic potato salad/mayo; cream filled pastries	- MCC foodborne illness - heat-stable enterotoxin - quick onset sxs within 6 hrs - frequently vomiting
  Bacillus cereus	Fried rice	- Two forms: 1 - heat-stable enterotoxin = vomiting (2–3 hrs post ingestion) 2- heat-labile enterotoxin = diarrhea (6–14 hrs after ingestion)
  Botulism	Home canned food; honey	- Heat-labile toxin - Sxs: DIPLOPIA; Ptosis; DESCENDING PARALYSIS; FLOPPY BABY SYNDROME
  Clostridium perfringens	Undercooked or sitting out too long (buffet) sausages/meats	- Heat resistant spore ingested → enterotoxin - Cramps and watery diarrhea
  ETEC (enterotoxigenic E. coli)	Food from street vendors; water/ice cubes in Mexico, Latin America, southern Asia, Africa	- MCC TRAVELER’S diarrhea - heat-labile & heat-stable toxins - Prophylactic abx not recommended; but should take 2 tabs of busmuth subsalicylate 4x a day preventatively - Tx: cipro or azithro
  Clostridium difficile Aka Pseudomembranous Enterocolitis	Occurs after antibiotics (clindamycin, cephalosporins, amoxicillin); Toxin A & B produced	- MCC bacterial/secretory diarrhea in inpatients - Dx: C. diff toxin immunoassay - Tx: oral vancomycin 1st line (old tx was flagyl) - Complication: Toxic megacolon
  Vibrio cholera	Contaminated food/water; associated w/shellfish/raw oysters	- Enterotoxin mediated Rice water diarrhea (copious amounts, very watery) = large volume loss → hypokalemic hypochloremic acidosis (loss of K+ & bicarb in feces) - Tx: IVF, doxycycline, zinc
  Listeria	Cold meats, soft cheeses	- Increases risk for preterm delivery = pregnant pts should avoid these foods
  Aeromonas hydrophila	Summer; untreated drinking water from well or spring	- Lactobacillus (probiotic) decreases duration
  Scombroid	Heat stable toxin found in tuna, mahi mahi (dolphin fish), mackerel - Hawaii, Florida	- Heat-stable toxins - caused by not properly refrigerating fish = sharp, metallic, bitter/peppery taste to fish - HISTAMINE LIKE REACTION sxs = flushed, headache, cramps, vomiting, diarrhea, bronchospasm or hypotension - Tx: H1 blockers (diphenhydramine) & H2 blockers (ranitidine)
  Ciguatera	Reef fish = grouper, barracuda, king fish, red snapper → when these fish eat a dinoflagellate	- Heat-stable neurotoxin - Acts on sodium channels = Neuro, Cardio, GI sxs - Sxs: paresthesias, ataxia, bradycardia, HOT/COLD REVERSAL → these sxs can actually last for years! ⇒ absolute abstinence from alcohol, seafood, and nuts until all sxs resolve and then 3–6 months after - Tx: H1/H2 blockers; amitriptyline; mannitol for severe sxs no longer recommended
- **Parasitic Diarrhea**
  [Table t9-jetem-6-2-sg8]
  **Table t9-jetem-6-2-sg8:**
  Parasite	Source	Other “BizzBuzz”
  Entamoeba Histolytica	Parasitic infection from contaminated food/water	- CNS, cardiac, pulmonary sxs after travel - Associated w/liver abscess, fatal cerebral amebiasis - Cystic and trophozoite phases - St. Petersburg, Russia has ongoing outbreak - All should be treated w/metronidazole + paroMOMycin (can be given to pregnant pts)
  Giardia lamblia	Mountain streams/beavers; spread via fecal-oral or anal sex	- Backpackers’ diarrhea - travel to Colorado or Russia - Steatorrhea (foul smelling fatty stool); bloating - Recurrent associated w/IgA deficiency - **Dx: stool antigen test** (NOT stool ova/parasite); stool will have cysts/trophozoites - Tx: metronidazole
  Cryptosporidium	Protozoan parasite	- MCC chronic diarrhea in AIDS pts - Tx: for HIV pts, HAART therapy (once CD4 >100 sxs might resolve) otherwise Tx with azithromycin & Paromomycin
  Isospora belli aka Cystoisospora	Coccidian parasite in subtropical regions	- Classic ex: HIV positive M from Haiti - Oocysts in stool - Acid-fast staining of fecal specimen - **eosinophilia** - Tx: TMP-SMX (or pyrimethamine, nitrofurantoin, or furazolidone in pts with sulfa allergy)
  Cyclospora	Coccidial parasite in tropical and subtropical regions	- Affects immunocompromised and immunocompetent patients the same
  Necator americanus aka Hookworm	Larvae penetrate intact barefoot skin on contaminated soil	- Larvae penetrate skin, enter bloodstream, ascend the trachea, descend the esophagus, then turn into adults and attach to the mucosal wall - Anemia and protein deficiency - Tx: mebendazole or albendazole or pyrantel pamoate PLUS iron
  Enterobius vermicularis aka Pinworm	Outbreaks in schools/daycares	- Pruritus ani at night → scotch tape test - Tx: single dose of mebendazole or pyrantel pamoate (repeat in 2 weeks, treat all family members)
- AIDS diarrhea =cryptosporidium (MCC), CMV, mycobacterium avium; giardia, entamoeba, isospora, cyclospora, C. diff, salmonella, shigella, yersinia, campylobacter

#### RECTUM/ANUS

- Rectal Prolapse (aka Procidentia)
  ○ Full thickness prolapse = circular folds vs partial thickness prolapse = radial folds
  ○ Can be a sign of cystic fibrosis in kids
  ○ Tx: try REAL not artificial sugar to help reduce; adults might need surgery, kids expectant management
- Rectal Foreign Body
  ○ Complications include anorectal laceration, bowel wall hematoma or focal bowel ischemia, bowel perforation
  ○ Tx: attempt careful removal w/foley catheter, Surgery consult if free air; after removal the patient should be observed x12 hrs and have repeat imaging and sigmoidoscopy performed
- Anal Fissure
  ○ MCC PAINFUL rectal bleeding
    ■ Internal hemorrhoids = MCC painLESS rectal bleeding
    ■ Diverticulosis = MCC LGIB
  ○ Posterior Midline = normal location for fissure
  ○ Non-healing or non-midline fissures makes you concerned about cancer, HIV, Crohn’s, TB, or sexual abuse
  ○ Tx: WASH regimen
    ■ Warm water sitz baths, Analgesia with topical anesthetic or topical nitrates, Stool softeners, High fiber diet
- Hemorrhoids
  ○ MCC bright red blood per rectum
  ○ Risk factors = constipation, straining, pregnancy, portal HTN, low fiber diet
  ○ Sxs: anal pruritis
    ■ External hemorrhoids = below dentate line, painFUL thrombosis
    ■ Internal hemorrhoids = above dentate line, painLESS thrombosis
  ○ Tx: sitz baths, analgesics, stool softeners, high fiber diet, topical steroids, surgery; thrombosed hemorrhoids can be incised via elliptical incision if <48 hrs
- Anorectal abscess
  [Fig f15-jetem-6-2-sg8]
  ○ Perianal abscess can be incised and drained in ED
  ○ Ischiorectal & Perirectal abscess should be drained in OR (abscesses above dentate line drained in OR)
- Pilonidal Cyst
  ○ Presacral abscess from hair follicles that become indurated at the midline superior buttock crease
  ○ Risk factors = hairy men, poor hygiene
  ○ ALWAYS MIDLINE
  ○ Most common complication is recurrence - refer to surgeon for excision
- Proctitis
  ○ Inflammation of rectum
  ○ Commonly caused by sexually transmitted infection (STI) or radiation
  ○ Sxs: pruritis, hematochezia, rectal discharge, tenesmus, pain
- Anorectal Tumors
  ○ Anal canal neoplasms = proximal to dentate line, 80% of anorectal tumors, high-grade malignant potential and poor prognosis
    ■ Adenocarcinoma; mucoepidermoid carcinoma; malignant melanoma; Kaposi’s sarcoma; squamous cell carcinoma; basaloid carcinoma; villous adenoma
  ○ Anal margin neoplasms = distal to dentate line, slower to metastasize, good prognosis
    ■ Basal cell carcinoma; squamous cell carcinoma; Bowen’s disease; extramammary Paget disease; giant solitary trichoepithelioma
